# Variability in the Population Diffusion Patterns of SARS‐CoV‐2 by Exposure Setting and Its Roles in Driving Epidemic Dynamics

**DOI:** 10.1111/irv.70125

**Published:** 2025-06-01

**Authors:** Chin Pok Chan, Ngai Sze Wong, Tsz Ho Kwan, Eng Kiong Yeoh, Shui Shan Lee

**Affiliations:** ^1^ S.H. Ho Research Centre for Infectious Diseases The Chinese University of Hong Kong Hong Kong China; ^2^ Stanley Ho Centre for Emerging Infectious Diseases The Chinese University of Hong Kong Hong Kong China; ^3^ JC School of Public Health and Primary Care The Chinese University of Hong Kong Hong Kong China; ^4^ Centre for Health Systems & Policy Research The Chinese University of Hong Kong Hong Kong China

**Keywords:** epidemic, outbreak dynamics, SARS‐CoV‐2, transmission cluster, virus exposure

## Abstract

**Background:**

Identifying transmission events that trigger epidemic spread is paramount for informing outbreak control. This study characterised the population diffusion patterns of SARS‐CoV‐2 across exposure settings and evaluate their ramifications in epidemic growth.

**Methods:**

In Hong Kong, COVID‐19 clusters delineated through case‐based surveillance during the pandemic period were classified into eight exposure settings: residence, home gathering, neighbourhood, workplace (office)/school, workplace (non‐office), daily activity, social activity and healthcare. Diffusion patterns characterised by outbreak size, speed and likelihood of spillover (cases seeding a new cluster) were compared among settings. With different clusters emerging, the lagged effect on effective reproduction number (*R*_*t*_) was evaluated.

**Results:**

Between January 2020 and January 2022, some 2800 clusters involving 14,202 cases were identified over five epidemic waves precipitated by outbreaks occurring in daily activity (wave I/III), social activity (wave II/IV) and neighbourhood (wave V—Omicron). Adjusted for variations by epidemic wave, the largest and fastest spread was observed in neighbourhood, averaging a size of 11.9 and daily generation of 1.18 cases per cluster. Spillover was the most common for social activity clusters with each of which normally breeding 3.73 onward clusters, compared to 0.18 for residential clusters. A cluster emerging in neighbourhood, social activity and daily activity was estimated to raise the *R*_*t*_ by 0.021–0.025, 0.013–0.024 and 0.008–0.015, respectively, on the ensuing 7 days.

**Conclusions:**

Neighbourhood and social activity outbreaks were inclined to induce epidemic spread, warranting the need for prioritised mitigation and targeted implementation of precautionary measures during both epidemics and peak season of respiratory infection.

## 1 Background

Driven by transmission heterogeneity, the setting of virus exposure had played a pivotal role in modulating the COVID‐19 outbreak dynamics [1]. Notably, the transmission dynamics of SARS‐CoV‐2 was shown to vary across settings. For example, outbreaks occurring in healthcare facilities [2] and nightlife activities [3] had featured a longer duration of propagation and a more extensive scale of virus spread, respectively. However, the characterisation of propagation was usually restricted to each specific setting. Regarding their reverberation on the broader epidemic dynamics, evidence has been lacking to date. Early in the COVID‐19 pandemic, disproportionate spread associated with long‐distance travel [4], congregate living setting [5] and party [6] was observed preceding the peak of an epidemic wave. This suggested that the proliferation of virus transmissions in certain settings could have possibly introduced radical changes to the epidemic trajectory. By monitoring the whereabouts of transmission chains, the identified trend could conceivably provide early warnings for epidemic escalation. Complementing the use of effective reproduction number (*R*_*t*_), the availability of such an indicator could also provide context for the emerging outbreak and inform the type of intervention required to counter the growing epidemic [7].

Towards an enhanced outbreak preparedness and response, understanding the common characteristics of transmission events that triggers epidemic spread is paramount. In Hong Kong, a massive cluster involving over 700 persons associated with SARS‐CoV‐2 transmissions in multiple dance clubs was demonstrated to have inflamed a new wave of infections [8]. A modelling study had also shed light on the unignorable effect of shorter generation interval on the epidemic spread of SARS‐CoV‐2 [9]. Elsewhere, a group of individuals who frequented nightclubs and bars was also found to have caused outbreaks in 16 associated venues and expedited community transmission as supported by phylogenetic evidence [10]. This collection of instances has implicated that the potential for a localised outbreak to induce territory‐wide spread could depend on the diffusion characteristics of the virus in the population, as reflected in its cluster size, speed of propagation as well as propensity for spillover to other settings [11]. With consideration of these three attributes, this study aims to characterise the population diffusion patterns of SARS‐CoV‐2 throughout the pandemic period in Hong Kong, uncover its association with different exposure settings, and evaluate their ramifications in epidemic growth.

## 2 Methods

### 2.1 Data Source

The ‘COVID‐19 surveillance line list’ which documented the cases confirmed by polymerase chain reaction (PCR) in Hong Kong formed the core dataset for analysis. Accessed at intervals from the Centre for Health Protection (CHP) of the HKSAR government, the dataset details the socio‐demographics of each case and their epidemiological characteristics, including the date of report and symptom onset, case classification (imported, local, close contact of imported case and close contact of local case) and involvement in transmission cluster. Another clinical dataset was retrieved from the Hospital Authority, which managed all public hospitals where COVID‐19 inpatient care was provided territory‐wide. For each confirmed case with quantitative PCR results targeting the E gene and available before or at most 2 days after isolation/hospitalisation, the record with the highest viral load was matched to the line list by a pseudo code. Access to the two datasets was approved by the respective authorities. Ethical approval was also obtained from the Joint CUHK‐NTEC Clinical Research Ethics Committee (CREC ref. no. 2023.006).

### 2.2 Classification of Exposure Setting

Generally, exposure setting refers to the environment where the case was potentially exposed to the pathogen. In this study, it was determined by the whereabouts of the clustered SARS‐CoV‐2 transmissions. According to the available description, each identified cluster was classified into one of the eight undermentioned categories of exposure setting in reference to our epidemiology research [12]. ‘Residence’ referred to any form of shared living environments, whereas ‘home gathering’ applied to the context where two or more individuals meeting at home typically in the relationship of couples or close relatives. ‘Neighbourhood’ pertained to those small communities which encompassed individuals living nearby such as apartment buildings. ‘Workplace (office)’ and ‘school’ were combined as a single category as the number of school‐based clusters were small (*n* = 18) due to the suspension of face‐to‐face teaching, and also given the shared close contact characteristics. ‘Workplace (non‐office)’ was set as another category to account for the heterogeneity in workplace environment. ‘Daily activity’ covered venues where day‐to‐day activities were taking place like public transportation and market, whereas ‘social activity’ referred to those events gathering people for socialising purpose. ‘Healthcare’ clusters consisted of outbreaks stemming from hospital and long‐term care facilities. Differentiated by the duration, frequency of contact and nature of interaction, the characteristics of close contact for each category were listed in Supplementary material 1.

### 2.3 Data Analysis

Spanning from the circulation of wild‐type SARS‐CoV‐2 to the early spread of the Omicron variant, cases reported between 23 January 2020 and 31 January 2022 were analysed. To maintain comparability, cases reported thereafter were excluded as contact tracing was severely hampered by the drastic epidemic growth in February 2022. Socio‐demographic and epidemiological characteristics of all the cases were first summarised using descriptive statistics. For a sharper distinction of outbreak dynamics, the entire COVID‐19 epidemic was divided into epidemic waves, with the conclusion of each indicated by the absence of clustered cases. At individual level, the weekly variations in exposure setting of SARS‐CoV‐2 was depicted. To avoid overrepresentation of transmission clusters, subclusters (clusters comprising a subset of cases from another cluster) were eliminated. Cases belonging to more than one clusters were double counted in the analyses.

‘Outbreak size’ was measured based on the total number of cases reported in a cluster. To account for overdispersion, the data was fitted to a negative binomial distribution. Adjusted for variations by epidemic wave, its association with exposure setting was assessed using negative binomial regression, taking residential clusters as the reference group. The presence of moderation effect by the index cases' Ct value as a proxy of SARS‐CoV‐2 viral load was further determined. As the minimum size of a cluster was defined as two in this study, the value of cluster size was transformed by subtracting two from each to match with the support on {0, 1, 2 … …} in a negative binomial distribution. Back‐transformation was done by adding a constant value of two in the computation of ratio for comparison with the reference group. ‘Speed of propagation’ was defined as the average number of cases occurred per day as the cluster progressed. Equivalently, its association with exposure setting and the moderation effect by viral load was determined using linear regression.

‘Spillover transmission’ referred to the occasion when a clustered case seeded another cluster of infections in a new setting. To assess the spillover likelihood in different settings, the transmission chains of SARS‐CoV‐2 was reconstructed. The cases' sequence of infection was first delineated within each cluster based on their order of symptom onset or the reporting date for asymptomatic cases. For each pair of clusters intertwined by the presence of a mutual case, the one that harboured the mutual case as an ‘index case’ (the earliest onset in the cluster) was designated to be the succeeding cluster, or otherwise the preceding cluster. Connections between each pair of linked clusters were amalgamated to inform the cascade of transmission as illustrated in Supplementary material 2. By exposure setting, the respective proportion of clusters that had led to spillover transmission or terminated a cascade was distinguished. With the application of graph theory, the ‘outdegree’, which indicated the number of onward clusters generated by the cases in a cluster, was also measured and compared among settings using multiple linear regression [13].

To analyse the association between exposure setting and epidemic dynamics, the time‐varying *R*_*t*_ was estimated for the local transmission of SARS‐CoV‐2 following Thompson et al. [14]. Imported cases giving rise to each wave, those occurring in the intermission period, and local cases reported between 1 and 28 February were re‐introduced to the working dataset for a realistic computation. Assuming a gamma distribution for the serial intervals of SARS‐CoV‐2 (mean 4.75 days; standard deviation [SD] 4.07 days), the *R*_*t*_ was modelled based on a 7‐day window using the R package ‘EpiEstim’ [15]. To gauge the daily change in *R*_*t*_ and reduce random noises, *R*_*t*_ was differenced to its first order and applied with a moving average filter for 3 days. Using distributed lag models, the transformed value of *R*_*t*_ was assessed against the emergence of clusters in different settings. A 6‐day lag was allowed presuming that the ripple effect of a cluster on epidemic dynamics lasts up to the 7th day of its emergence, equivalent to the average duration of a cluster. To avoid confounding by the changing control measures, the COVID‐19 stringency index reflecting the stringency of local policy responses was further controlled in the model [16]. Overall, a more positive lagged effect implied a stronger tendency for that type of outbreak to induce accelerated community spread.

Sensitivity analysis was conducted to account for the possibility of presymptomatic transmission. Assuming a gamma distribution for the incubation period of SARS‐CoV‐2 (mean 4.5 days; SD 2.23 days), the date of infection for each symptomatic case was inferred and used to reconstruct the transmission cascade in 100 simulations [17]. The resulting impact on spillover transmission was re‐examined. The estimation of *R*_*t*_ was also re‐run based on serial interval distributions derived from different epidemic periods to account for the bias possibly induced from the use of a fixed distribution [18].

R (version 4.2.0) was used to perform all statistical analyses. Cytoscape (version 3.9.1) was employed for the conduction of outdegree analyses. All statistical tests were two‐tailed and significance was denoted by *p* < 0.05.

## 3 Results

### 3.1 Characteristics of COVID‐19 Cases and Clusters

Between 23 January 2020 and 31 January 2022, totally 14,202 COVID‐19 cases were reported to the health authority. With a median age of 42 years (interquartile range [IQR] 29–58 years), half of them were female (51.8%). Symptoms such as cough and fever were present in two thirds (65.5%), while the median Ct value was estimated to be 22.2 (IQR 17.8–27.9). With 27.2% of the cases imported from places outside Hong Kong, 5.9%, 22.8% and 44% were close contacts of imported cases, local cases and their close contacts, respectively (Table 1).

**TABLE 1:** Socio‐demographic and epidemiological characteristics of COVID‐19 cases.

Variables	*n* (%)
**Sex**	
Male	6850 (48.2)
Female	7352 (51.8)
**Age (in years) (median, IQR)**	42 (29–58)
18 or below	925 (6.5)
19–29	2443 (17.2)
30–39	2667 (18.8)
40–49	2472 (17.4)
50–64	3388 (23.9)
65 or over	2307 (16.2)
**Presence of COVID‐19 symptom(s)**	
Yes	9309 (65.5)
No	4854 (34.2)
Unknown	39 (0.3)
**SARS‐CoV‐2 Ct value (median, IQR) (*n* = 11,125)**	22.2 (17.8–27.9)
**Case classification**	
Imported	3868 (27.2)
Close contact of imported case	842 (5.9)
Local	3239 (22.8)
Close contact of local case	6253 (44)
**Involvement in cluster**	
No	4397 (31)
Yes, involving in cluster(s) with imported cases only	1055 (7.4)
Yes, involving in local cluster(s)	8750 (61.6)

With the exclusion of 4397 non‐clustered cases (31%) and 1055 cases linked to an imported cluster (7.4%), some 8750 cases linking to a total of 2800 local clusters was retained for analysis. In general, a mean size and duration of 3.6 persons and 6.9 days was estimated for the clusters. As regards the classification by exposure setting, 1578 clusters were ascertained to be associated with virus exposure in residence (56.4%), followed by home gathering (*n* = 532, 19%), daily activity (*n* = 255, 9.1%), workplace (office) (*n* = 121, 4.3%), workplace (non‐office) (*n* = 100, 3.6%), social activity (*n* = 86, 3.1%), neighbourhood (*n* = 68, 2.4%), healthcare (*n* = 60, 2.1%) and school (*n* = 18, 0.6%).

### 3.2 Variations in SARS‐CoV‐2 Exposure Setting by Epidemic Wave

**FIGURE 1:**
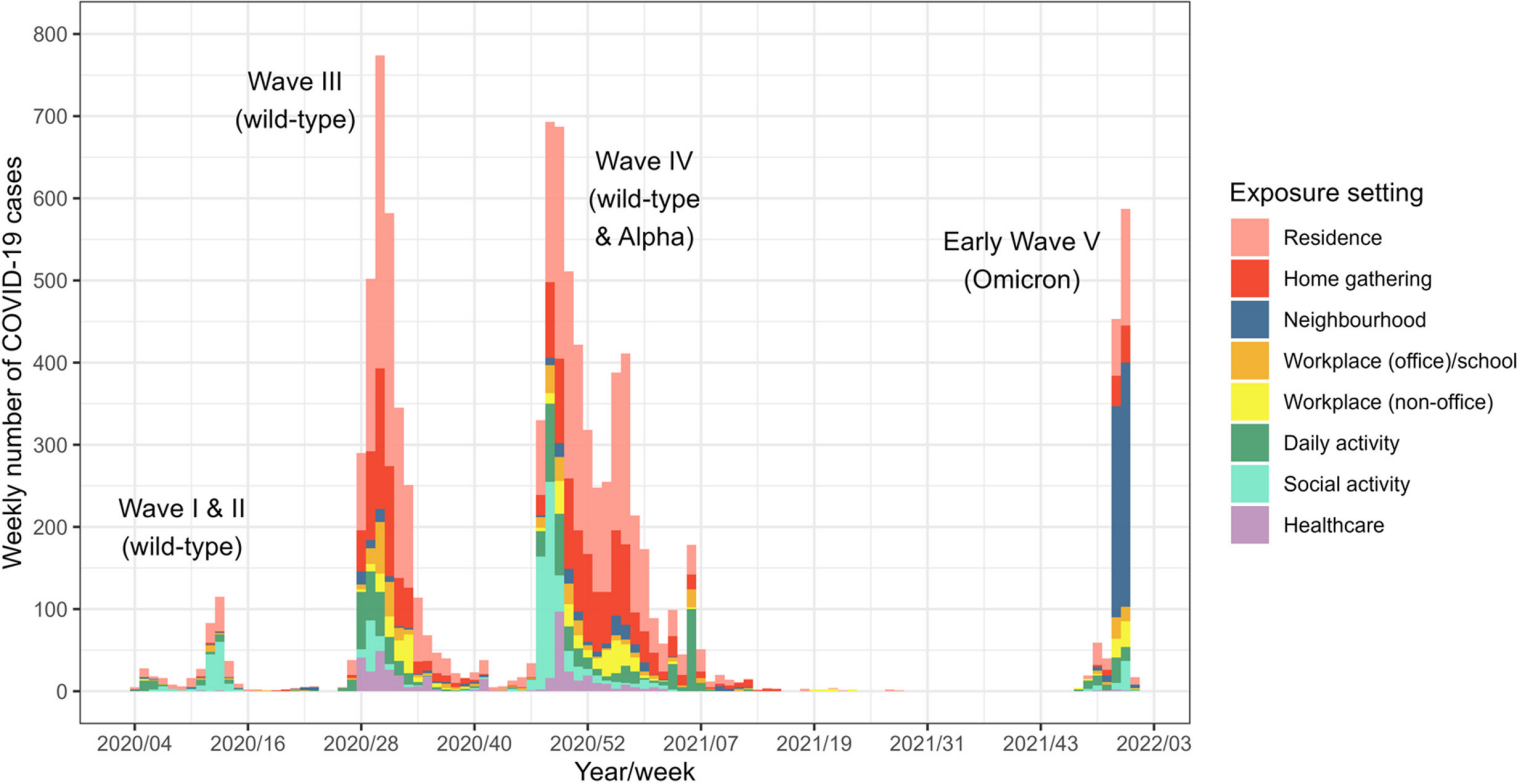
Temporal variations in exposure setting of SARS‐CoV‐2 by epidemic wave. The weekly number of COVID‐19 cases associated with clusters emerging in different exposure settings is depicted based on the date of symptom onset. In each epidemic wave, the dominating SARS‐CoV‐2 variant(s) is/are remarked in parentheses.

Five epidemic waves were distinguished with the onset of each led by different types of clusters (Figure 1). During wave I (weeks 4–9 of 2020), exposure to the wild‐type SARS‐CoV‐2 had mainly arisen from daily activities. However, as cases continued to accumulate in the population, outbreaks had more prominently occurred in the setting of social activity (bar and band) in wave II (weeks 10–23 of 2020). Initially, in wave III (weeks 24–43 of 2020), a few clusters comprising > 10 cases acquiring SARS‐CoV‐2 through daily activities (restaurant) had contributed to a more extensive spread in the community. Alongside sustained transmissions occurring in healthcare facilities, over 750 cases were reported weekly at the peak of this wave. Wave IV (week 44 of 2020 to week 19 of 2021) was composed of three consecutive sub‐waves provoked by clusters emerging in social activities (dance club), home gatherings and daily activities (gymnasium). With sporadic exposure observed in residences at the tail of wave IV, widespread transmissions of SARS‐CoV‐2 Omicron in several neighbourhoods had caused the eruption of wave V (week 51 of 2021 and onwards) after a 4‐month intermission period.

### 3.3 Outbreak Size, Speed of Propagation and Their Associations With Ct Value

Based on descriptive statistics, a larger cluster size (6.5 persons) was noted in the early wave V compared to the pre‐Omicron waves (3.2–3.5 persons). After controlling for variations by epidemic wave, all non‐residential clusters were found to be significantly larger than those occurring in residences based on results of negative binomial regression (Table 2). By transforming the regression coefficient into ratio for comparison with the reference group (a cluster emerging in residence during wave I/II with a Ct value of 21 for the index case), social activity, neighbourhood and healthcare clusters were estimated to be 2.27 (95% CI 1.87, 2.88), 2.15 (95% CI 1.73, 2.83) and 1.73 times (95% CI 1.45, 2.18) as large as the reference cluster. For other types of clusters, the adjusted ratio ranged from 1.12 to 1.31.

**TABLE 2:** Multivariable regression on outbreak size, speed of propagation and moderation of SARS‐CoV‐2 viral load by exposure setting.

Variables	Outbreak size (negative binomial model)			Speed of propagation^a^ (linear model)		
	Descriptive mean (SD)	Adjusted ratio [95% CI]^b^, ^c^	With interaction term: adjusted ratio [95% CI]^b^, ^c^	Descriptive mean (SD)	Adjusted B [95% CI]^c^	With interaction term: adjusted B [95% CI]^c^
**Epidemic wave**						
Wave I/II	3.52 (6.8)	1.00	1.00	0.46 (0.43)	Ref	Ref
Wave III	3.27 (3.68)	1.21 [1.05, 1.51]**	1.20 [1.05, 1.50]**	0.54 (0.44)	0.17 [−0.07, 0.41]	0.18 [−0.06, 0.43]
Wave IV	3.47 (10.67)	1.21 [1.05, 1.51]**	1.21 [1.05, 1.52]**	0.58 (0.51)	0.20 [−0.04, 0.44]	0.21 [−0.03, 0.45]
Early wave V	6.5 (33.65)	1.39 [1.13, 1.89]***	1.37 [1.13, 1.86]***	0.97 (2.26)	0.53 [0.27, 0.80]***	0.54 [0.28, 0.81]***
Intermission period	2.71 (0.76)	1.11 [0.90, 3.68]	1.12 [0.90, 3.68]	0.43 (0.29)	0.03 [−0.87, 0.92]	0.07 [−0.83, 0.96]
**Type of exposure setting**						
Residence	2.74 (1.09)	1.00	1.00	0.55 (0.45)	Ref	Ref
Home gathering	3.42 (1.97)	1.12 [1.08, 1.16]***	1.13 [1.01, 1.36]*	0.56 (0.44)	0.01 [−0.07, 0.09]	0.12 [−0.18, 0.41]
Neighbourhood	11.93 (54.71)	2.15 [1.73, 2.83]***	11.30 [3.46, 43.77]***	1.18 (3.63)	0.63 [0.43, 0.82]***	1.91 [1.15, 2.67]***
Workplace (office)/school	3.69 (4.71)	1.15 [1.07, 1.25]***	2.76 [1.43, 7.65]***	0.56 (0.42)	−0.02 [−0.16, 0.13]	−0.09 [−0.65, 0.48]
Workplace (non‐office)	4.67 (7.58)	1.31 [1.19, 1.48]***	3.70 [1.66, 11.97]***	0.57 (0.41)	−0.03 [−0.19, 0.13]	−0.12 [−0.76, 0.51]
Daily activity	3.55 (6.87)	1.13 [1.08, 1.20]***	1.05 [0.94, 1.35]	0.62 (0.58)	0.05 [−0.06, 0.15]	0.01 [−0.40, 0.42]
Social activity	9.97 (42.83)	2.27 [1.87, 2.88]***	1.26 [0.93, 3.62]	0.79 (0.97)	0.23 [0.05, 0.41]*	0.25 [−0.47, 0.97]
Healthcare	7.23 (10.94)	1.73 [1.45, 2.18]***	5.23 [2.04, 17.88]***	0.66 (0.68)	0.12 [−0.09, 0.33]	0.59 [−0.11, 1.30]
**Index case's Ct value (Ct)**	—	0.998 [0.997, 0.999]**	1.00 [0.998, 1.002]	—	−0.006 [−0.011, −0.001]*	−0.004 [−0.010, 0.003]
**Interaction terms**						
Residence*Ct	—	—	1.00	—	—	Ref
Home gathering*Ct	—	—	0.989 [0.942, 1.076]	—	—	−0.005 [−0.017, 0.008]
Neighbourhood*Ct	—	—	0.896 [0.886, 0.934]**	—	—	−0.058 [−0.091, −0.025]***
Workplace (office)/school*Ct	—	—	0.898 [0.887, 0.935]***	—	—	0.003 [−0.021, 0.028]
Workplace (non‐office)*Ct	—	—	0.90 [0.887, 0.942]***	—	—	0.004 [−0.023, 0.031]
Daily activity*Ct	—	—	1.052 [0.947, 1.329]	—	—	0.002 [−0.016, 0.020]
Social activity*Ct	—	—	1.288 [0.943, 4.336]	—	—	0 [−0.034, 0.033]
Healthcare*Ct	—	—	0.901 [0.887, 0.958]*	—	—	−0.023 [−0.057, 0.010]

Abbreviations: B, unstandardised coefficient; CI, confidence interval; SD, standard deviation.

*p < 0.05; **p < 0.01; ***p < 0.001.

^a^ Measured by dividing the number of cases reported by the total duration of a cluster.

^b^ Back‐transformed ratio of the predicted cluster size to the reference group (a cluster emerging in residence during Wave I/II with a Ct value of 21 for the index case).

^c^ Adjusted for all listed variables.

Similarly, a higher propagation speed was acknowledged within clusters in early wave V (generation of 0.97 case vs 0.46–0.58 cases per day). Accounting for such heterogeneity, a higher speed of propagation was still observed within neighbourhood (adjusted B 0.63 [95% CI 0.43, 0.82]) and social activity clusters (adjusted 0.23 [95% CI 0.05, 0.41]) with an estimate of 1.18 and 0.79 cases generated per cluster per day. This was in contrast to clusters associated with other settings, within which an average of 0.55–0.66 cases was produced per day.

Concerning the role of index cases' viral load, virus spread in clusters provoked by individuals with a higher viral load (lower Ct value) was evaluated to be both significantly larger (adjusted ratio 0.998 [95% CI 0.997, 0.999]) and faster (adjusted B −0.01 [95% CI −0.01, −0.002]). When Ct value was introduced to the model as an interaction term with exposure setting, such amplifying effect was revealed to be particularly pronounced in the context of neighbourhood spread. A higher index case's viral load appeared to have played a significant role in both extending (adjusted ratio 0.896 [95% CI 0.886, −0.934]) and accelerating (adjusted B −0.058 [95% CI −0.091, −0.025]) the virus spread in neighbourhood. A similar moderation effect of viral load on cluster size was also present in the workplace (office) (adjusted ratio 0.898 [95% CI 0.887, 0.935]), workplace (non‐office) (adjusted ratio 0.90 [95% CI 0.887, 0.942]) and healthcare setting (adjusted ratio 0.901 [95% CI 0.887, 0.958]).

### 3.4 Likelihood of Spillover by Exposure Setting

**FIGURE 2:**
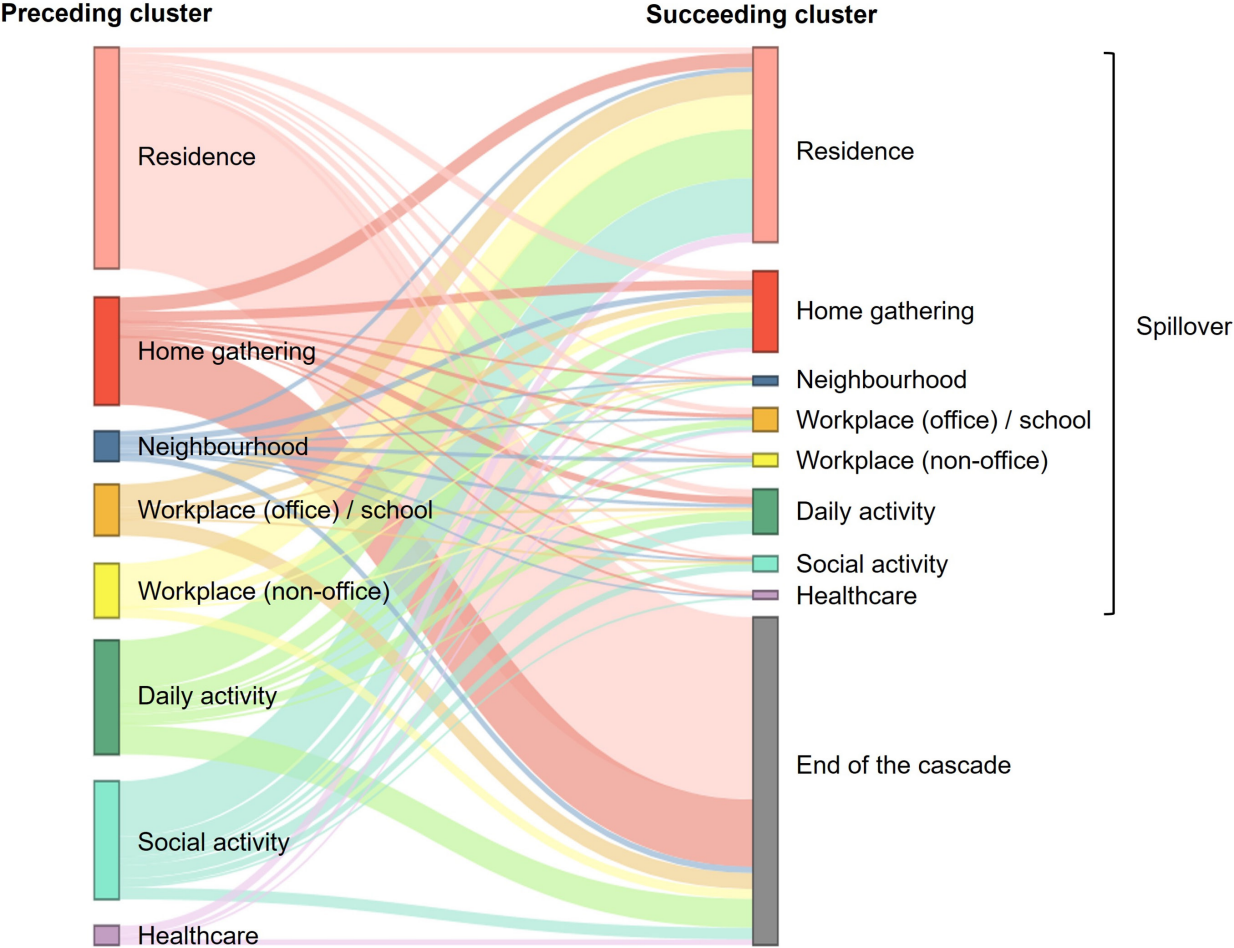
Propagation pattern of COVID‐19 clusters by exposure setting. The width of the connecting bands corresponds to the frequency of SARS‐CoV‐2 propagation from one type of cluster to another (spillover) or towards the end of the cascade. Cases in a (preceding) cluster could possibly generate more than one onward (succeeding) cluster and thus giving rise to multiple connecting bands.

Based on 1579 clusters inferred to have linked to at least one other cluster by the presence of a mutual case, totally 375 transmission cascades were reconstructed between wave I and early wave V (Supplementary material 3–6). By analysing the characteristics of 1411 edges representing the pattern of propagation, most edges departing from outbreaks occurring in residence (359/436, 82.3%) and home gathering (133/212, 62.7%) were directed towards the end of the cascade (Figure 2). In contrast, spillover to other settings was most frequently observed in social activity clusters covering 90.1% (210/233) of the outgoing edges, followed by workplace (non‐office) (82.2%, 88/107), neighbourhood (79.7%, 47/59), daily activity (74.7%, 168/225), healthcare (71.1%, 27/38) and workplace (office) clusters (68.3%, 69/101). Among the 765 identified spillover events, the most common propagations were from social activity (*n* = 109, 14.2%) and daily activity (*n* = 96, 12.5%) to residence. From the result of sensitivity analysis, the simulated proportion of spillover remained largely similar on average across exposure settings (Supplementary material 7).

**FIGURE 3:**
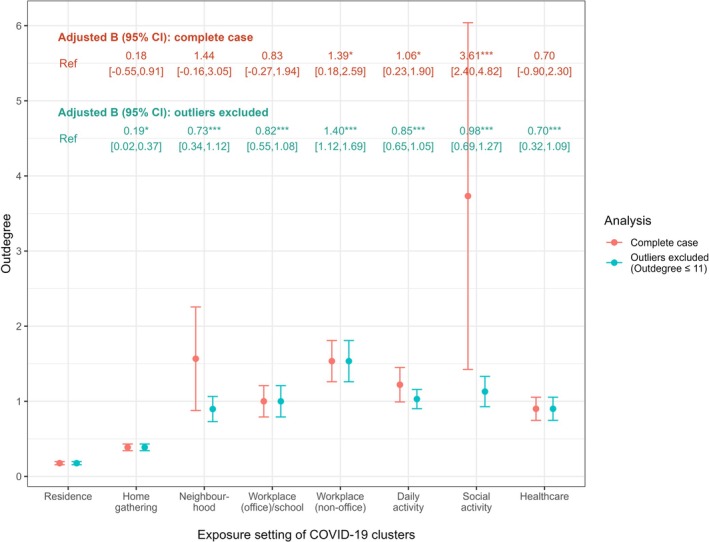
Outdegree of transmission clusters associated with different exposure settings. For analysis with outliers excluded, clusters with an outdegree of 19 or above were removed from the working dataset. The mean outdegree for both analysis scenarios were presented above each error bar, with the range of which representing the standard error. Epidemiologic waves were adjusted in the linear regression analysis, the results of which were presented at the top of the figure. B = unstandardised coefficient; CI = confidence interval; *p < 0.05; **p < 0.01; ***p < 0.001.

Showcasing the number of onward clusters generated by cases in a specific cluster, the outdegree was measured to be the highest for outbreaks occurring in social activities (3.73 onward clusters), followed by those associated with neighbourhood (1.57), workplace (non‐office) (1.53) and daily activities (1.22) (Figure 3). Among these exposure settings, however, only social activity (adjusted B 3.61 [95% CI 2.40, 4.82]), workplace (non‐office) (adjusted 1.39 [95% CI 0.18, 2.59]) and daily activity (adjusted B 1.06 [95% CI 0.23, 1.90]) demonstrated a significantly higher outdegree in comparison to clusters taking place in residence (0.18 onward clusters). Notably, the social activity clusters that triggered wave II (bar and band) and wave IV (dancing club) had resulted in an outdegree of 19 and 129, respectively. An outdegree of 21 was also noted for the neighbourhood cluster that initiated wave V. By excluding these three outlying cases together with another daily activity cluster surfacing towards the end of wave IV (outdegree 27), outbreaks occurring in all non‐residential settings had displayed a significantly higher outdegree compared to those linking to residence.

### 3.5 Lagged Effects on COVID‐19 Epidemic Dynamics

**FIGURE 4:**
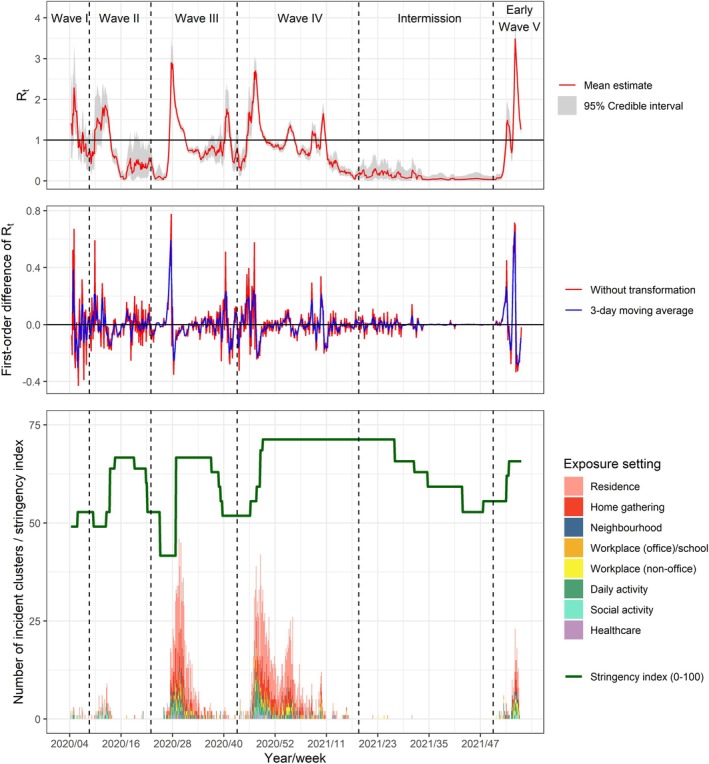
Effective reproduction number of SARS‐CoV‐2 and daily incidence of transmission clusters by exposure setting. The modelled effective reproduction number of SARS‐CoV‐2, its first‐order difference, the daily number of clusters emerging in different exposure settings and stringency index throughout the five epidemic waves are depicted. A higher stringency index reflects the implementation of more stringent public health measures, including but not limited to workplace closure, restriction on public gathering and travel ban.

Generally, the modelled *R*_*t*_ aligned well with the observed dynamics of COVID‐19 outbreaks (Figure 4). At the peak of waves I to V, the mean *R*_*t*_ was estimated to be 2.28, 1.85, 2.87, 2.70 and 3.48, respectively. With *R*_*t*_ transformed to its first‐order difference and applied with a 3‐day moving average, a cluster emanated from the neighbourhood setting was gauged to have accelerated the epidemic to the largest extent, controlling for the stringency index (Table 3). From the results, an expected increase in *R*_*t*_ by 0.023 (95% CI 0.006, 0.040), 0.024 (95% CI 0.007, 0.041), 0.025 (95% CI 0.009, 0.041), 0.021 (95% CI 0.005, 0.037), 0.025 (95% CI 0.010, 0.041) and 0.022 (95% CI 0.007, 0.038) was observed on the 2nd to 7th day of its emergence. Similar associations were observed in social activity clusters with an estimated 0.013 (95% CI 0.001, 0.026), 0.024 (95% CI 0.012, 0.037) and 0.017 (95% CI 0.005, 0.029) increase of *R*_*t*_ on the 5th to 7th day. For daily activity clusters, a smaller increment in *R*_*t*_ ranging from 0.008 to 0.015 had also resulted following its emergence on the 3rd to 6th day. An opposite effect was however suggested for clusters associated with workplace (office)/school, such that a decrease in *R*_*t*_ by 0.016–0.020 was estimated on the 4th to 6th day of its onset. An equivalent decline had also resulted in clusters arising in workplace (non‐office) on the 7th day (−0.017 [95% CI −0.030, −0.003]). For the emergence of residential, home gathering and healthcare clusters, a significant lagged effect on the epidemic was not detected.

**TABLE 3:** Distributed lag model for SARS‐CoV‐2 effective reproduction number and the emergence of clusters in different exposure settings.

Variables^a^	Lag weights						
	β_t_ [95% CI]	β_t‐1_ [95% CI]	β_t‐2_ [95% CI]	β_t‐3_ [95% CI]	β_t‐4_ [95% CI]	β_t‐5_ [95% CI]	β_t‐6_ [95% CI]
Residence	−0.001 [−0.003, 0.002]	−0.0002 [−0.003, 0.003]	−0.002 [−0.005, 0.001]	−0.002 [−0.005, 0.001]	−0.001 [−0.004, 0.002]	−0.001 [−0.004, 0.001]	−0.002 [−0.004, 0.001]
Home gathering	0.002 [−0.003, 0.007]	0.003 [−0.002, 0.008]	0.005 [−0.0002, 0.01]	0.004 [−0.001, 0.009]	0.001 [−0.004, 0.006]	−0.002 [−0.007, 0.003]	−0.004 [−0.009, 0.001]
Neighbourhood	0.011 [−0.006, 0.028]	0.023** [0.006, 0.040]	0.024** [0.007, 0.041]	0.025** [0.009, 0.041]	0.021* [0.005, 0.037]	0.025** [0.010, 0.041]	0.022** [0.007, 0.038]
Workplace (office)/school	0.012* [0.0001, 0.023]	0.006 [−0.005, 0.018]	−0.004 [−0.015, 0.008]	−0.016** [−0.028, −0.005]	−0.020*** [−0.031, −0.009]	−0.017** [−0.028, −0.006]	−0.004 [−0.015, 0.008]
Workplace (non‐office)	0.011 [−0.003, 0.024]	0.005 [−0.008, 0.019]	0.007 [−0.006, 0.020]	0.004 [−0.010, 0.018]	−0.003 [−0.016, 0.01]	−0.004 [−0.018, 0.009]	−0.017* [−0.030, −0.003]
Daily activity	0.002 [−0.004, 0.009]	0.004 [−0.002, 0.011]	0.008* [0.001, 0.014]	0.010** [0.004, 0.017]	0.015*** [0.008, 0.021]	0.008* [0.001, 0.014]	0.004 [−0.002, 0.010]
Social activity	0.006 [−0.007, 0.018]	0.012 [−0.002, 0.025]	0.005 [−0.008, 0.018]	0.008 [−0.005, 0.021]	0.013* [0.001, 0.026]	0.024*** [0.012, 0.037]	0.017** [0.005, 0.029]
Healthcare	−0.001 [−0.018, 0.015]	−0.007 [−0.024, 0.010]	−0.009 [−0.026, 0.007]	−0.011 [−0.028, 0.006]	−0.007 [−0.023, 0.009]	−0.002 [−0.018, 0.014]	0.009 [−0.007, 0.024]
Stringency index	−0.001 [−0.006, 0.004]	0.0003 [−0.006, 0.007]	0.003 [−0.004, 0.009]	0.001 [−0.005, 0.007]	−0.001 [−0.007, 0.005]	0.0004 [−0.006, 0.007]	−0.004 [−0.008, 0.001]

Abbreviation: CI, confidence interval.

*p < 0.05; **p < 0.01; ***p < 0.001.

^a^ Number of clusters emerging in different settings, stringency index and the lag weights of both were adjusted simultaneously.

From results of the sensitivity analysis, the assumed serial interval distribution appeared to be a good approximation of those at different periods for the estimation of *R*_*t*_ (Supplementary material 8). By rerunning the distributed lag model using different serial interval distributions, the estimated lagged effects remained largely similar (Supplementary material 9 and 10).

## 4 Discussion

By analysing the population diffusion pattern of SARS‐CoV‐2 in various outbreaks, this study substantiated the role of exposure setting as an important driver of epidemic dynamics. In light of the ensuing increase in *R*_*t*_, a preponderance of clustered transmissions revealed in neighbourhood and social environments was postulated to have triggered epidemic spread in the community. Their progression to epidemicity was also speculated to be through different mechanisms. Through the surveillance of exposure setting, the identified characteristics of virus propagation could lend support to a more precise formulation of mitigation measures in future epidemic.

Outbreaks stemming from neighbourhoods had demonstrated the highest potential to elicit widespread transmissions at community level. Transmitted through drainage pipelines in high‐rise buildings, the dissemination of virus which led to concurrent exposure in multiple households had likely contributed to the immense size and rapid development of outbreaks [19]. During the early wave V in Hong Kong, local lockdown was in place for 7 days when a single residential building had accumulated 20 cases [20]. With all residents confined in the same building serving as a densely connected network, those uninfected could be subject to recurring exposure from newly infected cases even after the index patient was isolated elsewhere [21]. The difficulty in removing the contaminated source completely from the building could challenge the interruption of transmission chains and hence accelerate the epidemic. With the need to trace a massive number of contacts made by successively infected residents, the omission of some secondary cases could also lead to unobserved transmissions in the community [22]. With less onward clusters being uncovered, this could also justify why a significantly higher outdegree had not resulted in neighbourhood outbreaks despite the enormous scale of spread.

The present study has also added that the scale and speed of neighbourhood transmission depends on the index patient's viral load. During the 2003 SARS outbreak, infected residents living closer to the index case were found to carry a higher viral load of SARS‐CoV [23]. More recently, a simulation‐based study also saw a positive correlation between aerosol SARS‐CoV‐2 viral load and attack rate among users in a shared environment [24]. The collection of evidence had given plausibility to the role of viral load in potentiating aerosol transmission, the route of which was deemed pre‐eminent in the neighbourhood setting.

Outbreaks emerging in social occasions were another instance characterised by the breadth and rapidity of virus propagation. Echoing the clustered transmissions unveiled in a wedding banquet [25] and karaoke rooms [26], this could be attributed to the ease of virus transmission driven by private interactions [27]. Typically characterised as a single‐day event, social activities also enable the encounter of people who do not meet regularly. With diseases spreading within the group, infected individuals could in turn pose as a repeated source of infection to their daily contacts [28]. This explains why spillover transmissions from social activity clusters were ubiquitous in contrast to other settings. Occasions like worship gathering also assemble people coming from diverse social backgrounds. In the presence of substantial network heterogeneity, this could also allow the diffusion of virus through the patrons' unique social networks [29]. The derivation of multiple transmission chains was likely behind its exceptional outdegree and ability to provoke epidemic escalation [30].

Outbreaks arising in daily activities were inferred to have driven a minor increase in *R*_*t*_. Sharing a similar spillover mechanism as to social activity clusters, it is anticipated that the lower transmissibility in settings like public transport had rendered it a decreased likelihood of prompting widespread transmissions [31]. Unusually, wave III was caused by several clusters occurring in restaurants. Noting the reduced stringency in that period, the lowering of control measures had likely provided fuel for the proliferation of these outbreaks [32].

The rest of the exposure settings had contributed less to the epidemic dynamics. While home gathering was a setting that became more visible only after the closure of socialising venues, workplace/school was almost the remaining environment that permitted social mixing when stringent measures were implemented [33]. With a limited size of spread within a network of regular contacts, the onset of associated outbreaks during the contraction phase of an epidemic could probably explain the resulting decrease in *R*_*t*_.

The observed heterogeneity among exposure settings was also suggested to be a cause of overdispersion in the broader epidemic context. Throughout the local epidemic, neighbourhood and social activity clusters had occurred at a relatively low frequency. Yet, these clusters had showcased the largest scale of transmission, highest outdegree and strongest lagged effects on the epidemic dynamics. This has proven the phenomenon of overdispersion at cluster level, where a small fraction of clusters had provoked a larger number of downstream infections. Consistent with a systematic review which found high overdispersion among non‐household contacts, this has reiterated the importance of targeting specific community settings for epidemic control [34].

Of note, several limitations were present in this study. Foremost, the COVID‐19 vaccination status of the cases was not considered in the analyses. With a lower perceived risk, vaccinated individuals could be more likely to patronise crowded venues and thus hampering the comparability of outbreak dynamics in different settings [35]. The effect was however anticipated to be insubstantial as vaccination requirement for entry to specific premises was not enforced throughout the study period. Moreover, a higher transmissibility of SARS‐CoV‐2 was also expected after the emergence of variants of concern in wave IV [36]. The increased immunity escape for Alpha and Omicron variants could lead to the generation of larger outbreaks in later waves [37], although the effect of which was statistically adjusted in the present analyses. In this connection, the observed variability in diffusion patterns shall remain valid for the examination of disease spread caused by the newer sub‐lineages of Omicron. The varying combinations of social distancing interventions in force could also shape population behaviours and contribute to the uneven distribution of virus exposure across settings [1]. Yet, the resembling patterns derived from multifaceted analyses did verify the roles of exposure setting in driving epidemic dynamics. The designation of index case for a cluster was also based on the date of symptom onset. This could lead to inaccurate assignment if a cluster was indeed engendered by asymptomatic cases, despite the rarity of asymptomatic transmission. The type of swab collected for quantitative PCR testing was also not available. This has prevented us from taking into account its potential confounding effects in the regression analysis. The preclusion of ‘unlinked cases’ which might actually belong to a cluster may also affect the delineation of transmission clusters as in other analyses of contact tracing data. Extrapolation of findings to other communities should also be cautioned as the context of social interaction in settings like home gathering could vary between cultures. Transmission heterogeneity driven by factors like adherence to mask‐wearing and ventilation could also exist within the same category of exposure setting. Further studies might be required to refine the classification of exposure settings and evaluate the contribution by these factors on transmission risks.

This study carried important implications on the control of respiratory epidemics. The common occurrence of spillover transmissions warranted the amelioration of case‐finding efforts against social activity outbreaks. Designating a lower contact threshold for quarantine and employing second‐degree contact tracing should be considered for an expanded testing coverage [38, 39]. To tackle neighbourhood outbreaks, the evaluation of viral load should also be contemplated for refining quarantine and isolation strategies. Upon the detection of high viral load among infected residents, immediate and overarching confinement measures are needed to restrain further spread outside the neighbourhood. The mitigation of outbreaks arising from non‐residential settings should also be prioritised over those occurring within households, as a majority of which appeared at the end of the virus propagation cascade. The study findings have also called for the timely and targeted implementation of precautionary measures, like mask‐wearing, in social activity venues for attenuating seasonal respiratory outbreaks in the post‐pandemic era [40]. At policy level, a relevant classification of exposure settings should also be developed and incorporated into the disease surveillance framework for an improved epidemic forecast and outbreak management.

## Author Contributions

Conceptualisation: Eng Kiong Yeoh, Shui Shan Lee; methodology: Chin Pok Chan, Tsz Ho Kwan; formal analysis and investigation: Chin Pok Chan; writing – original draft preparation: Chin Pok Chan; writing – review and editing: Chin Pok Chan, Ngai Sze Wong, Tsz Ho Kwan, Eng Kiong Yeoh, Shui Shan Lee; funding acquisition and resources: Eng Kiong Yeoh, Shui Shan Lee; supervision: Ngai Sze Wong, Shui Shan Lee.

## Conflicts of Interest

The authors declare no conflicts of interest.

### Peer Review

The peer review history for this article is available at https://www.webofscience.com/api/gateway/wos/peer‐review/10.1111/irv.70125.

## Supporting information


**Data S1** Category of exposure setting and the assumed characteristics of close contact.
Data S2 Schematic diagram for reconstruction of transmission cascade.
Data S3 Reconstructed transmission network of SARS‐CoV‐2 in wave I/II.
Data S4 Reconstructed transmission network of SARS‐CoV‐2 in wave III.
Data S5 Reconstructed transmission network of SARS‐CoV‐2 in wave IV.
Data S6 Reconstructed transmission network of SARS‐CoV‐2 in early wave V.
Data S7 Sensitivity analysis for spillover transmission based on transmission cascades reconstructed from the inferred dates of SARS‐CoV‐2 infection.
Data S8 Estimation of effective reproduction number based on different assumptions of serial interval distribution.
Data S9 Distributed lag model for SARS‐CoV‐2 effective reproduction number using the wave II serial interval distribution (mean = 5.5 days, SD = 2.4 days).
Data S10 Distributed lag model for SARS‐CoV‐2 effective reproduction number using the early wave V serial interval distribution (mean = 2.72 days, SD = 1.51 days).

## Data Availability

The dataset cannot be shared because the data are owned by third parties. Access to these data and permission could be inquired through the Hospital Authority and Department of Health, Hong Kong SAR Government.
