# Supplementary material for: Variability in the Population Diffusion Patterns of SARS‐CoV‐2 by Exposure Setting and Its Roles in Driving Epidemic Dynamics
Source: Influenza Other Respir Viruses. 2025 Jun 1;19(6):e70125. doi: 10.1111/irv.70125 (PMC12127217; doi:10.1111/irv.70125)
Supplement: Supplementary file 1 — Data S1 Category of exposure setting and the assumed characteristics of close contact. Data S2 Schematic diagram for reconstruction of transmission cascade. Data S3 Reconstructed transmission network of SARS‐CoV‐2 in wave I/II. Data S4 Reconstructed transmission network of SARS‐CoV‐2 in wave III. Data S5 Reconstructed transmission network of SARS‐CoV‐2 in wave IV. Data S6 Reconstructed transmission network of SARS‐CoV‐2 in early wave V. Data S7 Sensitivity analysis for spillover transmission based on transmission cascades reconstructed from the inferred dates of SARS‐CoV‐2 infection. Data S8 Estimation of effective reproduction number based on different assumptions of serial interval distribution. Data S9 Distributed lag model for SARS‐CoV‐2 effective reproduction number using the wave II serial interval distribution (mean = 5.5 days, SD = 2.4 days). Data S10 Distributed lag model for SARS‐CoV‐2 effective reproduction number using the early wave V serial interval distribution (mean = 2.72 days, SD = 1.51 days). [file IRV-19-e70125-s001.docx]

**Supplementary material 1. Category of exposure setting and the assumed characteristics of close contact**

| Category | Examples of setting | Duration  of contact | Frequency of contact | Nature of interaction |
| --- | --- | --- | --- | --- |
| Residence | Household, dormitory, hotel | Prolonged | Daily | Familiar |
| Home gathering | Gathering with persons regularly met at home like partners and relatives | Moderate/ prolonged | Repeated | Familiar |
| Neighbourhood | Housing estates and communal areas in the vicinity | Short | Repeated | Variable |
| Workplace (office)/school | Office, school | Moderate | Daily | Familiar |
| Workplace  (non-office) | Non-office settings including service industry and outdoor workplaces | Moderate | Daily | Familiar |
| Daily activity | Shopping, public transport, restaurant, personalised service | Short | Repeated | Casual |
| Social activity | Entertainment, party, worship | Moderate | One-off | Familiar |
| Healthcare | Hospital, clinic, long-term care facility | Variable | Variable | Variable |

**Supplementary material 2. Schematic diagram for reconstruction of transmission cascade**


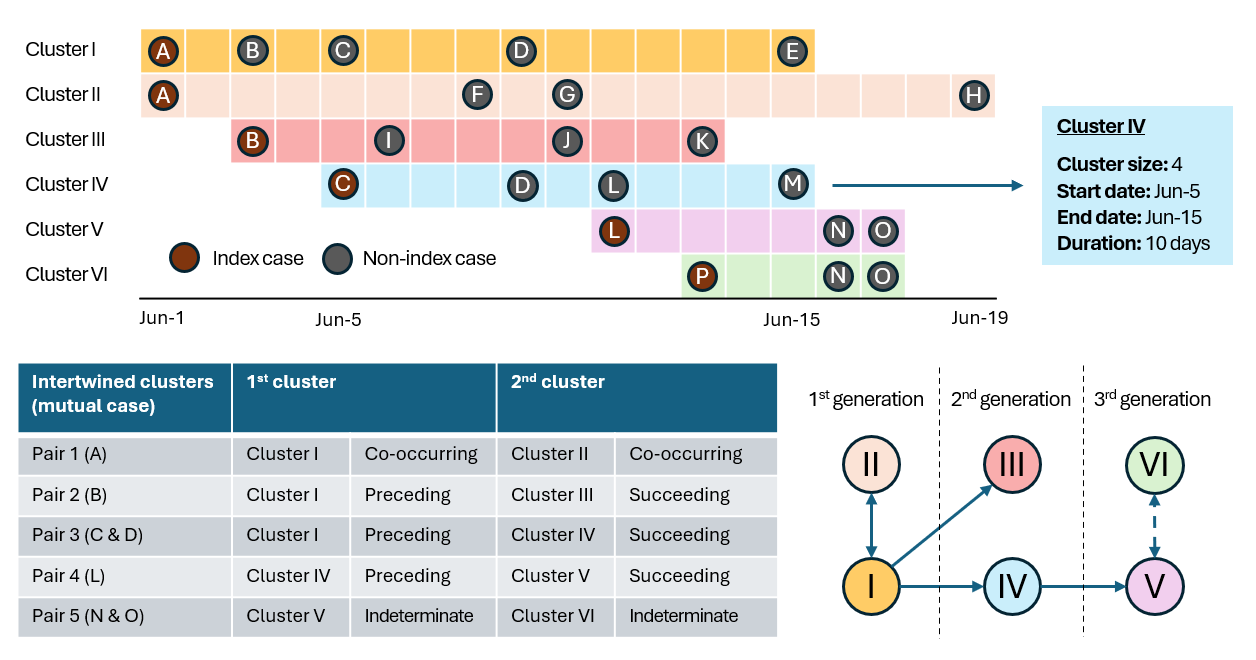


From the surveillance line list, clusters intertwined by the presence of a mutual case were first identified and grouped into the same block, such that each block consisted of all constituent clusters of a cascade. For each pair of intertwined clusters, the direction of propagation was deduced by the sequence of infection of the mutual case(s) in the two clusters. The cluster which harboured the mutual case as an index case would be designated as the “succeeding cluster”, or otherwise the “preceding cluster”. If the mutual case(s) was/were present as the index case in both clusters, the pair of clusters would be noted as “co-occurring”. If the mutual case(s) was/were present as a non-index case in both clusters, the direction of propagation would be classified as indeterminate. The resulting cascade of transmission for this group of intertwined clusters was illustrated at the bottom right corner. Only linkage with a deducible direction of propagation was included in the outdegree analyses.

**Supplementary material 3. Reconstructed transmission network of SARS-CoV-2 in Wave I/II**

**
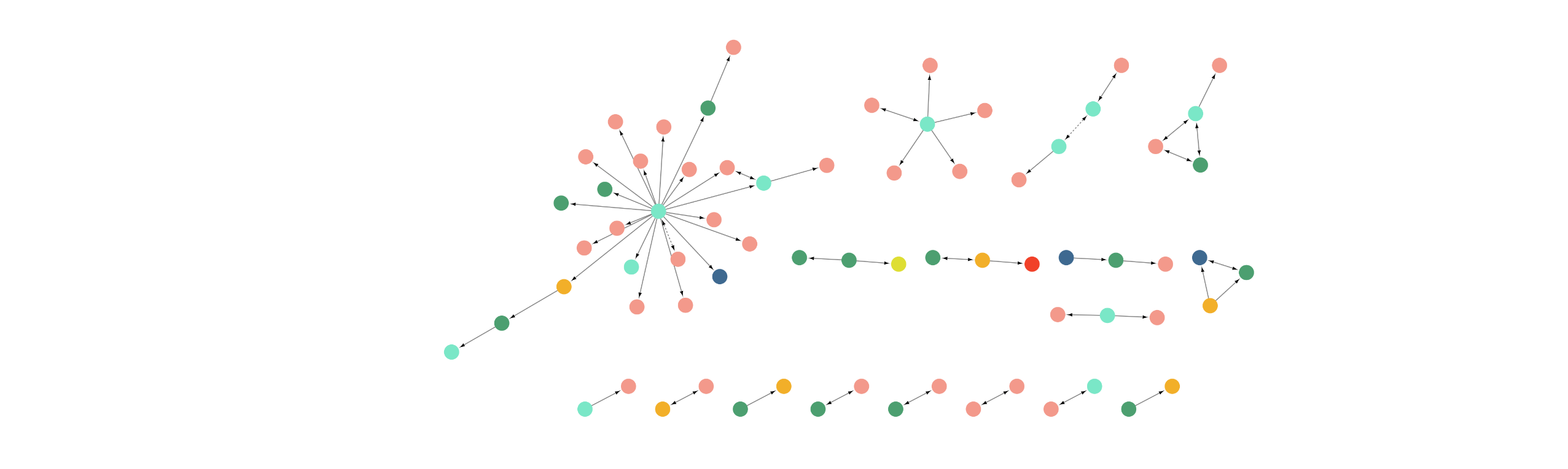
**

**Exposure setting**

Residence

Residence (non-co-living)

Neighbourhood

Workplace (office) / school

Workplace (non-office)

Daily activity

Social activity

Healthcare

**Type of linkage**

Preceding cluster Succeeding cluster

(known direction of propagation)

Co-occurring cluster Co-occurring cluster

(sharing the same index case)

Linked cluster Linked cluster

(unknown direction of propagation)

The above 17 transmission cascades were reconstructed from 70 transmission clusters. Clusters were linked by a total of 56 edges, of which 73.2% were directional and 23.2% conjoined clusters sharing the same index cases. The direction of cluster propagation was indeterminate in 3.6% of the edges.

**Supplementary material 4. Reconstructed transmission network of SARS-CoV-2 in Wave III**

**
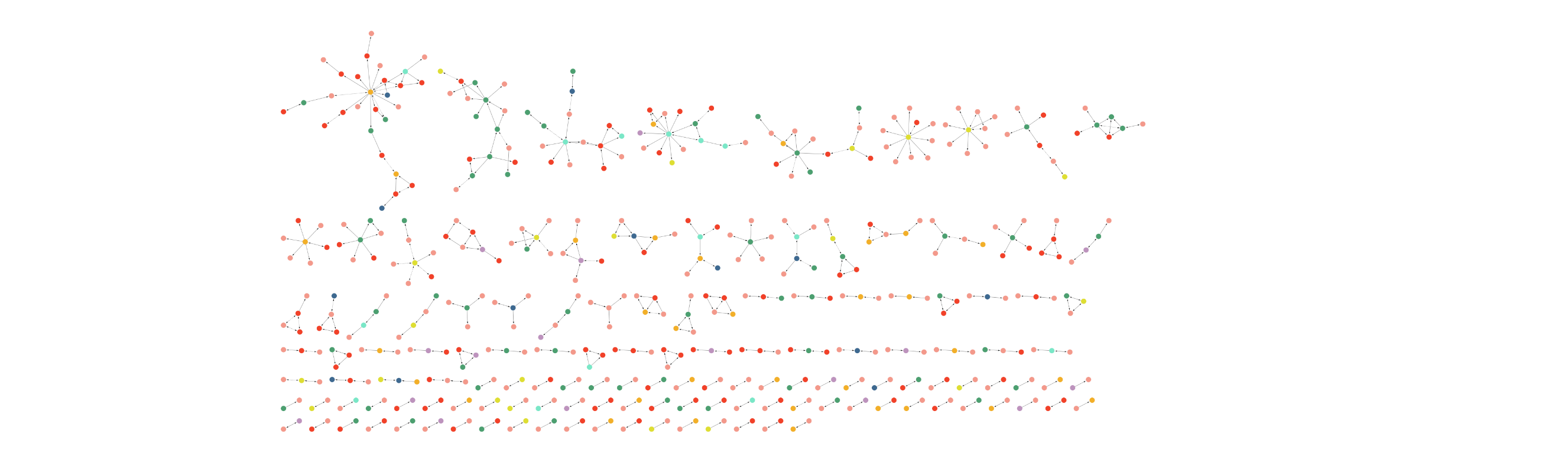
**

**Exposure setting**

Residence

Residence (non-co-living)

Neighbourhood

Workplace (office) / school

Workplace (non-office)

Daily activity

Social activity

Healthcare

**Type of linkage**

Preceding cluster Succeeding cluster

(known direction of propagation)

Co-occurring cluster Co-occurring cluster

(sharing the same index case)

Linked cluster Linked cluster

(unknown direction of propagation)

The above 136 transmission cascades were reconstructed from 488 transmission clusters. Clusters were linked by a total 394 edges, of which 48.7% were directional and 34.3% conjoined clusters sharing the same index cases. The direction of cluster propagation was indeterminate in 17% of the edges.

**Supplementary material 5. Reconstructed transmission network of SARS-CoV-2 in Wave IV**

**
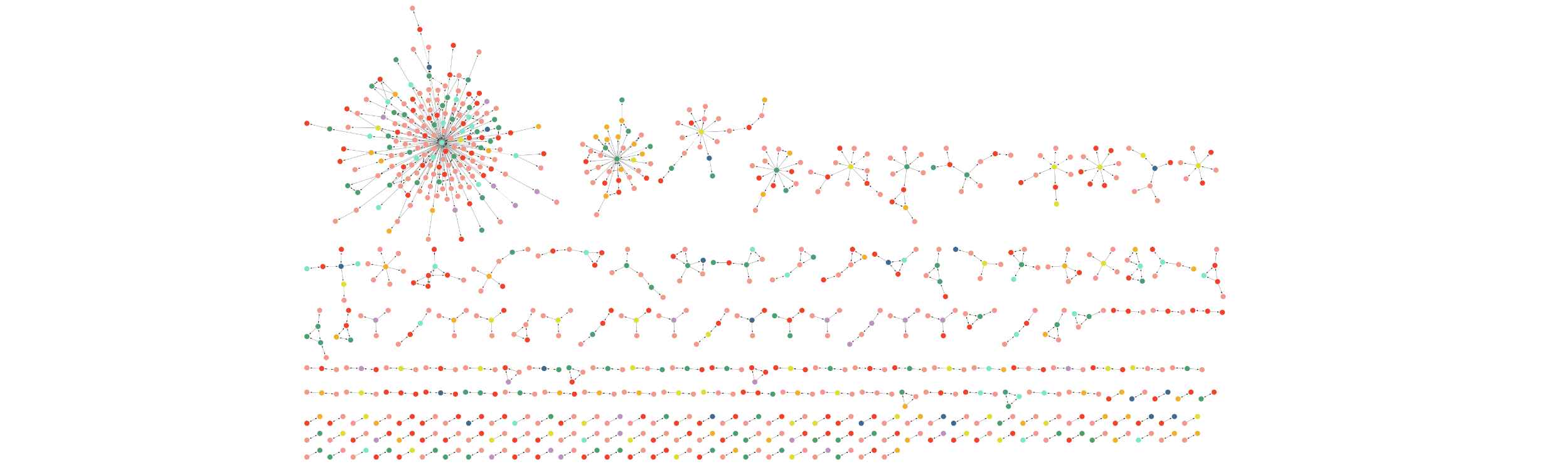
**

**Exposure setting**

Residence

Residence (non-co-living)

Neighbourhood

Workplace (office) / school

Workplace (non-office)

Daily activity

Social activity

Healthcare

**Type of linkage**

Preceding cluster Succeeding cluster

(known direction of propagation)

Co-occurring cluster Co-occurring cluster

(sharing the same index case)

Linked cluster Linked cluster

(unknown direction of propagation)

The above 210 transmission cascades were reconstructed from 874 transmission clusters. Clusters were linked by a total 744 edges, of which 59% were directional and 29.4% conjoined clusters sharing the same index cases. The direction of cluster propagation was indeterminate in 11.6% of the edges.

**Supplementary material 6. Reconstructed transmission network of SARS-CoV-2 in early Wave V**

**
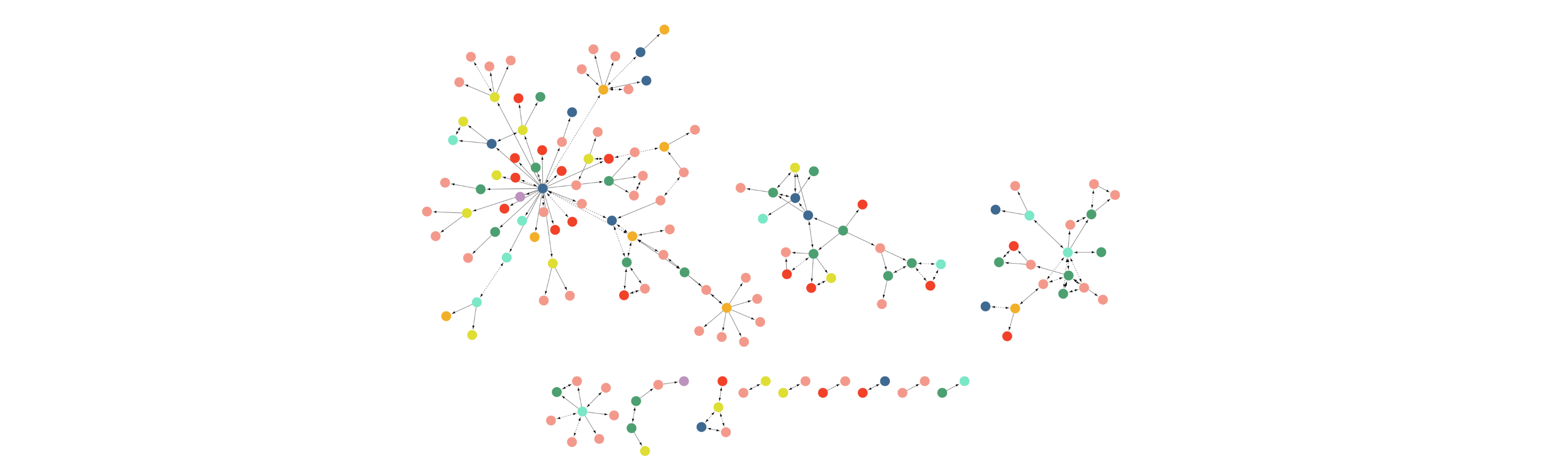
**

**Exposure setting**

Residence

Residence (non-co-living)

Neighbourhood

Workplace (office) / school

Workplace (non-office)

Daily activity

Social activity

Healthcare

**Type of linkage**

Preceding cluster Succeeding cluster

(known direction of propagation)

Co-occurring cluster Co-occurring cluster

(sharing the same index case)

Linked cluster Linked cluster

(unknown direction of propagation)

The above 12 transmission cascades were reconstructed from 147 transmission clusters. Clusters were linked by a total 161 edges, of which 57.8% were directional and 20.5% conjoined clusters sharing the same index cases. The direction of cluster propagation was indeterminate in 21.7% of the edges.

**Supplementary material 7. Sensitivity analysis for spillover transmission based on transmission cascades reconstructed from the inferred dates of SARS-CoV-2 infection**

**
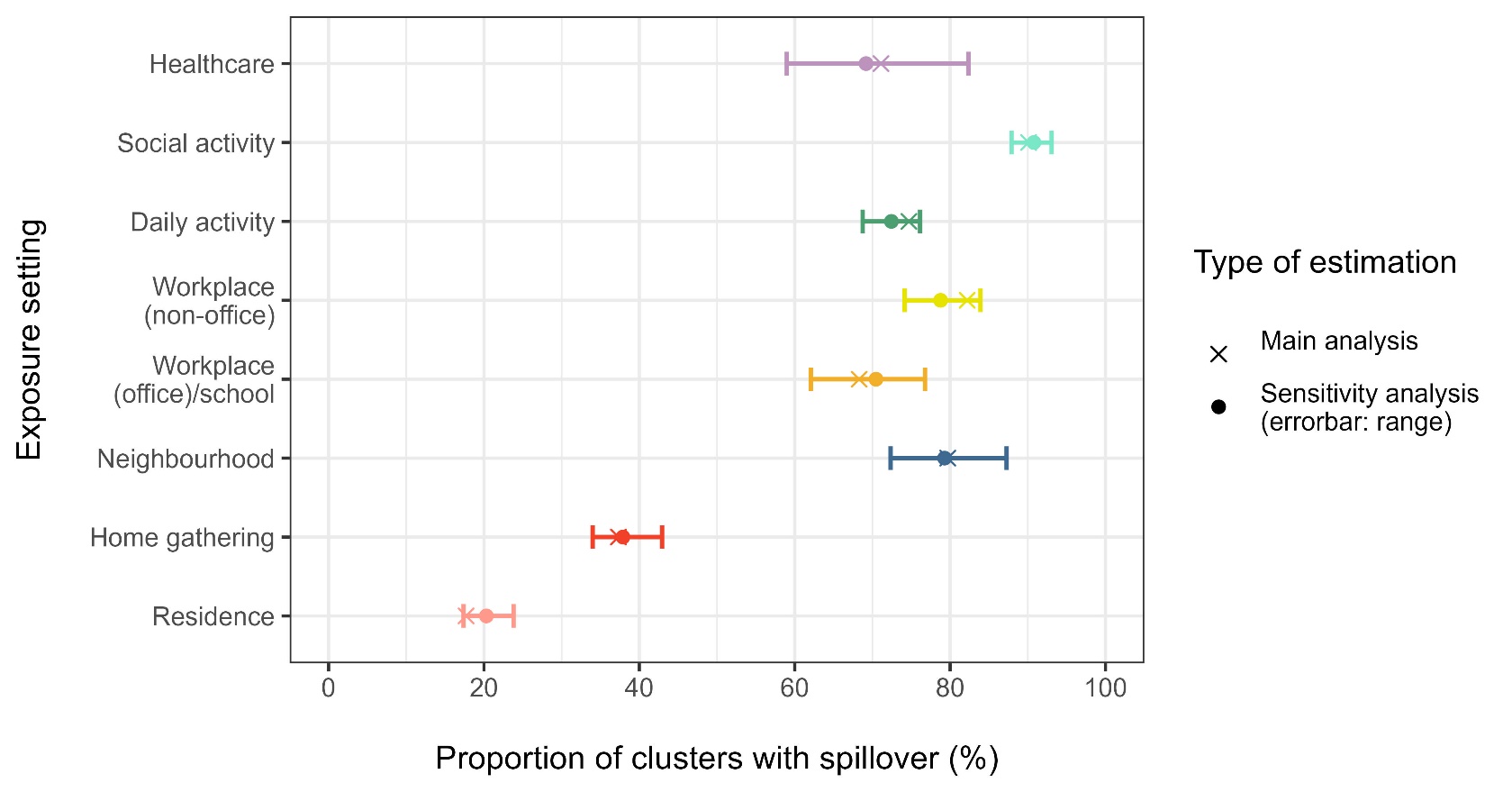
**

To account for the possibility of presymptomatic transmission, a sensitivity analysis was conducted such that a case is allowed to infect others in the same cluster before symptom onset. Assuming a gamma distribution for the incubation period of SARS-CoV-2 (mean 4.5 days; SD 2.23 days) [1], the date of infection for each symptomatic case was inferred. By running 100 simulations using the inferred dates, the proportion of different clusters with spillover transmission was examined in the reconstructed transmission cascades. The error bar indicates the range of values resulting from the simulation.

**Supplementary material 8. Estimation of effective reproduction number based on different assumptions of serial interval distribution**


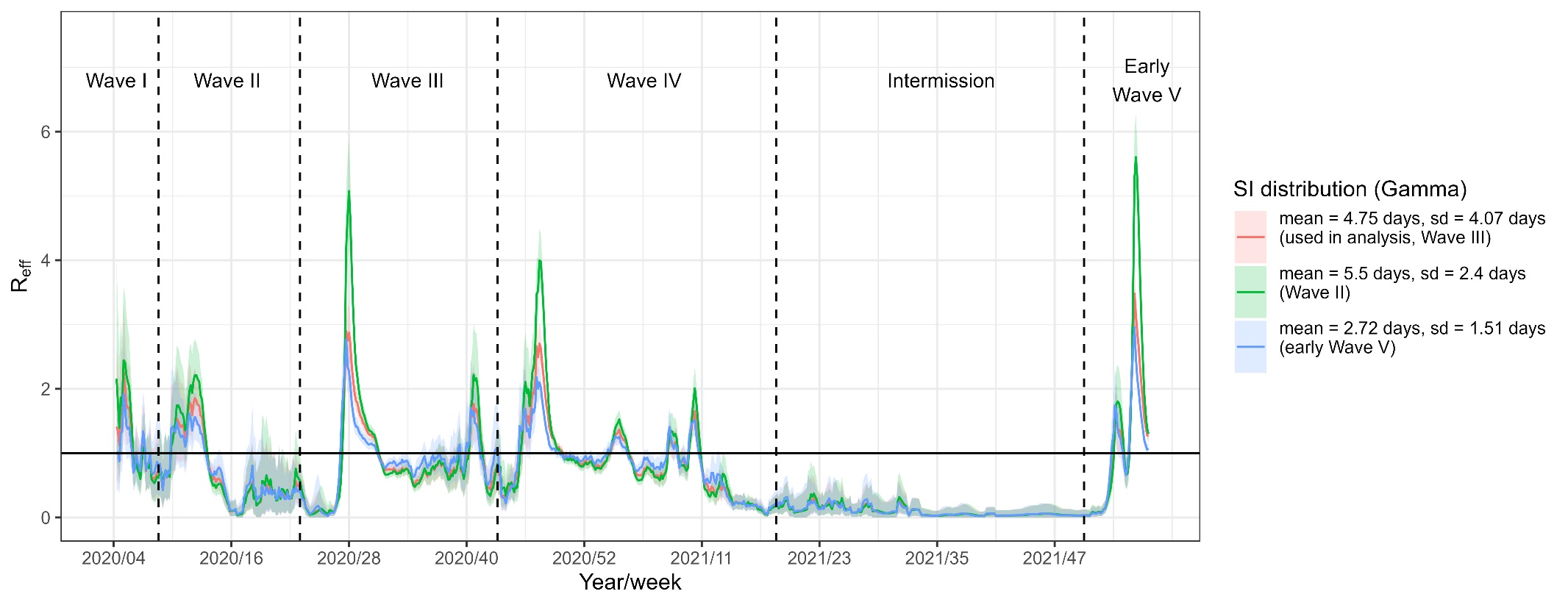


SARS-CoV-2 effective reproduction number estimated using serial interval (SI) distributions derived from the period of Wave II (green) [2], III (red) [3] and early wave V (blue) [4] were compared for examining the sensitivity. Given the similar curves resulting in Wave I-II derived from the Wave II (green) and III (red) distributions, and those resulting in early Wave V derived from the Wave III (red) and early Wave V (blue) distributions, the Wave III distribution was employed to approximate the SI at different periods in the main analysis.

**Supplementary material 9. Distributed lag model for SARS-CoV-2 effective reproduction number using the Wave II serial interval distribution (mean = 5.5 days, SD = 2.4 days)**

|  | **Lag weights** |  |  |  |  |  |  |
| --- | --- | --- | --- | --- | --- | --- | --- |
| **Variables ^†^** | **β_t_**  **[95%CI]** | **β_t-1_**  **[95%CI]** | **β_t-2_**  **[95%CI]** | **β_t-3_**  **[95%CI]** | **β_t-4_**  **[95%CI]** | **β_t-5_**  **[95%CI]** | **β_t-6_**  **[95%CI]** |
| Residence | -0.001 [-0.005, 0.004] | -0.002 [-0.007, 0.003] | -0.004. [-0.009, 0] | -0.003 [-0.008, 0.001] | -0.001 [-0.006, 0.004] | -0.002 [-0.007, 0.003] | -0.004 [-0.008, 0.001] |
| Home gathering | 0.007 [-0.001, 0.014] | 0.008* [0.001, 0.016] | 0.011** [0.003, 0.019] | 0.007 [-0.001, 0.015] | -0.002 [-0.011, 0.006] | -0.009* [-0.017, -0.001] | -0.011** [-0.019, -0.003] |
| Neighbourhood | 0.028* [0.001, 0.055] | 0.051*** [0.024, 0.077] | 0.057*** [0.03, 0.084] | 0.051*** [0.025, 0.077] | 0.047*** [0.021, 0.072] | 0.051*** [0.025, 0.076] | 0.044*** [0.019, 0.069] |
| Workplace (office)/school | 0.01 [-0.008, 0.028] | 0.002 [-0.016, 0.021] | -0.015 [-0.033, 0.003] | -0.032*** [-0.05, -0.014] | -0.029** [-0.047, -0.011] | -0.016 [-0.034, 0.002] | 0.006 [-0.013, 0.024] |
| Workplace (non-office) | 0.015 [-0.006, 0.037] | 0.001 [-0.02, 0.023] | 0.007 [-0.014, 0.028] | 0.006 [-0.015, 0.028] | -0.013 [-0.034, 0.008] | -0.017 [-0.038, 0.004] | -0.034** [-0.055, -0.013] |
| Daily activity | 0.009 [-0.002, 0.019] | 0.009 [-0.001, 0.02] | 0.016** [0.006, 0.027] | 0.016** [0.006, 0.027] | 0.02*** [0.01, 0.031] | 0.01 [0, 0.021] | 0.005 [-0.005, 0.015] |
| Social activity | 0.013 [-0.007, 0.033] | 0.018 [-0.003, 0.039] | 0.012 [-0.009, 0.033] | 0.022* [0.002, 0.043] | 0.036*** [0.016, 0.056] | 0.04*** [0.021, 0.06] | 0.023* [0.004, 0.043] |
| Healthcare | 0 [-0.026, 0.026] | -0.015 [-0.041, 0.012] | -0.025 [-0.051, 0.001] | -0.021 [-0.047, 0.006] | -0.005 [-0.031, 0.021] | 0.006 [-0.02, 0.032] | 0.03* [0.005, 0.055] |
| Stringency index | 0 [-0.008, 0.007] | 0.001 [-0.009, 0.012] | 0.006 [-0.004, 0.017] | 0.002 [-0.008, 0.012] | -0.004 [-0.014, 0.006] | -0.005 [-0.015, 0.005] | -0.001 [-0.009, 0.006] |

**^†^** Number of clusters emerging in different settings, stringency index and the lag weights of both were adjusted simultaneously

CI = confidence interval; *p < 0.05; **p < 0.01; ***p < 0.001

**Supplementary material 10. Distributed lag model for SARS-CoV-2 effective reproduction number using the early Wave V serial interval distribution (mean = 2.72 days, SD = 1.51 days)**

|  | **Lag weights** |  |  |  |  |  |  |
| --- | --- | --- | --- | --- | --- | --- | --- |
| **Variables ^†^** | **β_t_**  **[95%CI]** | **β_t-1_**  **[95%CI]** | **β_t-2_**  **[95%CI]** | **β_t-3_**  **[95%CI]** | **β_t-4_**  **[95%CI]** | **β_t-5_**  **[95%CI]** | **β_t-6_**  **[95%CI]** |
| Residence | -0.001 [-0.004, 0.002] | -0.001 [-0.004, 0.002] | -0.002 [-0.005, 0.001] | -0.002 [-0.005, 0.001] | -0.002 [-0.005, 0.001] | -0.002 [-0.005, 0.001] | -0.002 [-0.005, 0.001] |
| Home gathering | 0.001 [-0.004, 0.006] | 0.001 [-0.004, 0.006] | 0.003 [-0.002, 0.008] | 0.004 [-0.001, 0.009] | 0.003 [-0.002, 0.008] | 0.002 [-0.003, 0.007] | 0 [-0.005, 0.005] |
| Neighbourhood | 0.006 [-0.011, 0.023] | 0.017* [0, 0.034] | 0.018* [0.001, 0.035] | 0.022** [0.006, 0.038] | 0.021* [0.005, 0.037] | 0.026** [0.01, 0.043] | 0.021** [0.006, 0.037] |
| Workplace (office)/school | 0.017** [0.005, 0.029] | 0.012* [0.001, 0.024] | 0.002 [-0.009, 0.014] | -0.01 [-0.022, 0.001] | -0.015** [-0.027, -0.004] | -0.014* [-0.025, -0.002] | -0.004 [-0.016, 0.007] |
| Workplace (non-office) | 0.009 [-0.004, 0.023] | 0.006 [-0.008, 0.019] | 0.006 [-0.007, 0.02] | 0.006 [-0.008, 0.019] | 0.002 [-0.012, 0.015] | 0 [-0.013, 0.014] | -0.011 [-0.024, 0.002] |
| Daily activity | 0 [-0.007, 0.006] | 0.002 [-0.005, 0.009] | 0.003 [-0.003, 0.01] | 0.007* [0.001, 0.014] | 0.011** [0.004, 0.018] | 0.005 [-0.001, 0.012] | 0.002 [-0.005, 0.008] |
| Social activity | 0.002 [-0.011, 0.014] | 0.006 [-0.008, 0.019] | -0.002 [-0.016, 0.011] | -0.002 [-0.015, 0.011] | 0.004 [-0.009, 0.016] | 0.016* [0.003, 0.028] | 0.014* [0.002, 0.026] |
| Healthcare | -0.002 [-0.019, 0.014] | -0.003 [-0.019, 0.014] | -0.002 [-0.018, 0.015] | -0.004 [-0.021, 0.013] | -0.005 [-0.021, 0.011] | -0.003 [-0.02, 0.013] | 0.003 [-0.013, 0.019] |
| Stringency index | -0.002 [-0.007, 0.003] | 0.001 [-0.006, 0.007] | 0.002 [-0.004, 0.009] | 0.001 [-0.006, 0.007] | 0 [-0.006, 0.006] | 0.002 [-0.004, 0.009] | -0.005* [-0.01, -0.001] |

**^†^** Number of clusters emerging in different settings, stringency index and the lag weights of both were adjusted simultaneously

CI = confidence interval; *p < 0.05; **p < 0.01; ***p < 0.001

**References in the supplementary materials:**

1. Galmiche S, Cortier T, Charmet T, Schaeffer L, Chény O, von Platen C, et al. SARS-CoV-2 incubation period across variants of concern, individual factors, and circumstances of infection in France: a case series analysis from the ComCor study. Lancet Microbe. 2023 Jun;4(6):e409-e417. doi: 10.1016/S2666-5247(23)00005-8.
2. Ali ST, Chen D, Lau YC, Lim WW, Yeung A, Adam DC, et al. Insights into COVID-19 epidemiology and control from temporal changes in serial interval distributions in Hong Kong. Am J Epidemiol. 2024 Jul 16:kwae220. doi: 10.1093/aje/kwae220.
3. Kwok KO, Wei WI, Huang Y, Kam KM, Chan EYY, Riley S, et al. Evolving Epidemiological Characteristics of COVID-19 in Hong Kong From January to August 2020: Retrospective Study. J Med Internet Res. 2021 Apr 16;23(4):e26645. doi: 10.2196/26645.
4. Mefsin YM, Chen D, Bond HS, Lin Y, Cheung JK, Wong JY, et al. Epidemiology of Infections with SARS-CoV-2 Omicron BA.2 Variant, Hong Kong, January-March 2022. Emerg Infect Dis. 2022 Sep;28(9):1856-1858. doi: 10.3201/eid2809.220613.
